# Breastfeeding beliefs and experiences of African immigrant mothers in high‐income countries: A systematic review

**DOI:** 10.1111/mcn.12970

**Published:** 2020-03-05

**Authors:** Adefisayo O. Odeniyi, Nicholas Embleton, Lem Ngongalah, Wanwuri Akor, Judith Rankin

**Affiliations:** ^1^ Population Health Sciences Institute Newcastle University Newcastle upon Tyne UK; ^2^ Newcastle Neonatal Service Royal Victoria Infirmary Newcastle upon Tyne UK

**Keywords:** African immigrant, attitudes, beliefs, breastfeeding, infant feeding, knowledge

## Abstract

Breastfeeding provides optimal nutrition for the healthy growth of infants and is associated with reduced risks of infectious diseases, child and adult obesity, type 2 diabetes, and other chronic diseases. Migration has been shown to influence breastfeeding especially among migrants from low‐and‐middle‐income countries. This mixed‐methods systematic review aimed to identify, synthesise, and appraise the international literature on the breastfeeding knowledge and experiences of African immigrant mothers residing in high‐income countries. MEDLINE, CINAHL, Embase, PsychINFO, Scopus, and Web of Knowledge databases were searched from their inception to February 2019. Grey literature, reference, and citation searches were carried out and relevant journals hand‐searched. Data extraction and quality assessment were independently carried out by two reviewers. An integrated mixed‐methods approach adopting elements of framework synthesis was used to synthesise findings. The initial searches recovered 8,841 papers, and 35 studies were included in the review. Five concepts emerged from the data: (a) breastfeeding practices, showing that 90% of African mothers initiated breastfeeding; (b) knowledge, beliefs, and attitudes, which were mostly positive but included a desire for bigger babies; (c) influence of socio‐demographic, economic, and cultural factors, leading to early supplementation; (d) support system influencing breastfeeding rates and duration; and (e) perception of health professionals who struggled to offer support due to culture and language barriers. African immigrant mothers were positive about breastfeeding and willing to adopt best practice but faced challenges with cultural beliefs and lifestyle changes after migration. African mothers may benefit from more tailored support and information to improve exclusive breastfeeding rates.

## Graphical abstract

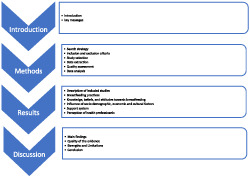

**Key messages**

- African mothers living in HICs consider breastfeeding as the natural and typical thing to do but tend to supplement with formula from as early as 3 months.
- Early supplementation with formula was associated with being in a high‐income country.
- Lifestyle changes after migration and the cultural beliefs about breastfeeding, including the perceived need to supplement breastfeeding with formula to achieve a bigger baby, were the main factors influencing exclusive breastfeeding rates.

## 1 INTRODUCTION

Breastfeeding provides optimal nutrition for infants for healthy growth and development and can be considered a low‐cost intervention. It is associated with much lower risks for infection and long‐term outcomes such as child and adult obesity, type 2 diabetes, and other chronic diseases (Binns, Lee, & Low, 2016). The World Health Organisation (WHO) recommends that infants be exclusively breastfed for at least the first 6 months of life (WHO, 2016). Despite this, the WHO still reports that, as of 2010, only 35% of infants worldwide were exclusively breastfed within the first 4 months of life (WHO, 2016). Breastfeeding has been recognised as beneficial not only to the infant but also to the mother as it is associated with a reduction in the development of various cancers (Collaborative Group on Hormonal Factors in Breast Cancer, 2002; Dewey, Heinig, & Nommsen, 1993; Labbok, 2001; Riman et al., 2002; Tung et al., 2003) and maternal depression (Mezzacappa, 2004). It is also associated with infant‐to‐mother bonding (Hart, Boylan, Carroll, Musick, & Lampe, 2003) and may aid quicker return to pre‐pregnancy weight (Rea, 2004).

There is evidence to show that breastfeeding is more widely practiced in low‐and‐middle income countries (LMICs) than it is in most high‐income countries (HICs) (Victora et al., 2016). A recent systematic review exploring the factors influencing exclusive breastfeeding (EBF) practices in LMICs showed that the existence of barriers to breastfeeding in these countries was insufficient to halt breastfeeding practices (Balogun, Dagvadorj, Anigo, Ota, & Sasaki, 2015). The study found that mothers living in these countries develop strategic plans alongside their inherent personal characteristics to ensure successful breastfeeding. This suggests that breastfeeding is considered by the mother to be an inherent part of an infant's health and growth in LMICs. Conversely, mothers from similar origins residing in HICs, who were faced with the same barriers such as maternal employment and insufficient supply of breast milk, were more likely to compromise previously held infant‐feeding beliefs and practices (Landolt & Wei Da, 2005; Ryan, 2007; Wall & José, 2004). This may be the result of prevalent breastfeeding practices observed in their host countries, such as the privacy of breastfeeding which reduced observational learning processes (Giles et al., 2007). Several studies have shown that migration from a lower income country into a HIC may have negative impacts on breastfeeding initiation, exclusivity, and duration (Dancel et al., 2015; Gibson‐Davis & Brooks‐Gunn, 2006; Harley, Stamm, & Eskenazi, 2007; Hawkins, Lamb, Cole, & Law, 2008; Nguyen et al., 2004; Textor, Tiedje, & Yawn, 2013; Twamley, Puthussery, Harding, Baron, & Macfarlane, 2011; Tyler, Kirby, & Rogers, 2014).

Several factors have been identified to influence breastfeeding practices, such as the mother's beliefs and knowledge about breastfeeding, the support available, the availability of formula milk alternatives, perception of cultural norms, and economic factors (Armstrong & Reilly, 2002; Baghurst et al., 2007; Bhopal, 2007; Dennis, 2006; Earle, 2002; Gallegos, Vicca, & Streiner, 2015; Howie, Forsyth, Ogston, Clark, & Florey, 1990; Meedya, Fahy, & Kable, 2010; United Nations Children's Fund, 2012). Earle (2002) found that many mothers made decisions about infant feeding practices long before conception and most of these decisions were based on hearsay, societal acceptability, and cultural beliefs. Such beliefs and assumptions held tenaciously create resistance to national and international recommendations on infant feeding (Kannan, Carruth, & Skinner, 1999).

Owing to the differences in breastfeeding beliefs and practices between those living in LMICs and HICs, there is evidence to show that immigrant mothers may or may not adopt practices of their host countries (Berry, 1997; Erten, van den Berg, & Weissing, 2018), despite the similarity of influential factors in both home and host countries (Gallegos et al., 2015; McFadden, Atkin, & Renfrew, 2014). A study conducted in Australia showed that African immigrant mothers maintained infant feeding practices from their countries of origin such as eating of special foods to aid milk production and enhance breastfeeding but also adopted some of the practices of the host country such as not breastfeeding in public places, which is contrary to practices in their home country (Gallegos et al., 2015). In the United States, breastfeeding is more commonly practiced among non‐Hispanic whites than most minority groups (Louis‐Jacques, Deubel, Taylor, & Stuebe, 2017), which is contrary to the United Kingdom, where breastfeeding rates are higher among ethnic minority mothers (McAndrew et al., 2012). Owing to this, an understanding of the factors that influence breastfeeding decisions and practices among different groups of mothers is necessary to understand why there may be differences in practices in different HICs, as well as offer adequate and relevant guidance to mothers regarding breastfeeding.

This mixed‐methods systematic review therefore aimed to explore the beliefs, attitudes, knowledge, and practices of African immigrant mothers who reside in HICs and how these may differ from the beliefs and practices in their home countries.

## 2 METHODS

A systematic review was carried out according to the Preferred Reporting Items for Systematic Review and Meta‐Analysis guidelines (Moher, Liberati, Tetzlaff, & Altman, 2010). A review protocol was developed detailing the processes and methods to be used in completing the review and was entered onto the National Institute for Health Research PROSPERO database.

### 2.1 Search strategy

A detailed search strategy with keywords and indexed terms was developed with assistance from an information specialist at Newcastle University. Databases searched included MEDLINE, Embase, PsychINFO, Cumulative Index to Nursing and Allied Health Literature, Scopus, Web of Knowledge, GreyNet, Proquest, and the Health Management Information Consortium. The Cochrane Database of Systematic Reviews, Database of Abstracts of Reviews of Effects, and the Health Technology Assessment databases were searched for relevant systematic reviews and reports. All databases were searched from their inception to November 2017. Key journals and conference proceedings from Breastfeeding Conferences and Conferences on Baby Friendly Initiative were hand‐searched from inception to present date. Database searches were completed in December 2017. Reference list searching and follow‐up citation searching of relevant articles and reviews identified were carried out and completed in February 2018. An updated search of the electronic databases was carried out in February 2019, and no additional relevant studies were identified.

### 2.2 Inclusion and exclusion criteria

Studies were included in the review if they met the following inclusion criteria: (a) primary research studies, (b) study participants were African mothers of childbearing age (16–45 years), who have migrated from an African country and are living in a HIC; (c) study was carried out in a HIC; (d) study was published in the English Language; and (e) study reported on any of the following outcomes: factors affecting choice of infant feeding such as beliefs, attitudes, facilitators, barriers, motivation or experience of breastfeeding, or breastfeeding practices such as initiation and duration of breastfeeding or weaning practices. Studies were excluded if participants were not clearly defined including definitions such as “Black,” “African‐American,” or “Negroid.” Immigration status of the mothers was not adequately explained or if human immunodeficiency virus‐positive mothers were the sole focus of the study due to the uncertainties around infant feeding practices and options among human immunodeficiency virus‐positive mothers (WHO et al., 2010).

### 2.3 Study selection

All records identified from electronic database screening were imported into Endnote X8 and deduplicated. Screening by titles and abstracts was carried out, with three authors (AO, NE, and JR) independently screening a 10% sample of the identified studies and AO completing the screening of the remaining 90%. The full texts of the potentially relevant studies were assessed for relevance with two authors independently assessing an initial 30% for eligibility: LN screened 20%, WA screened 10%, and AO screened all 30%. The remaining 70% full text studies were then checked for inclusion by one reviewer AO. Citation and reference searches of all included studies were completed by a single reviewer (AO), and the process was repeated for any additional studies eligible for inclusion.

### 2.4 Data extraction

Two data extraction tools were developed and piloted for this review, one for qualitative studies and the other for quantitative studies. All data extraction was done independently by two reviewers. One reviewer (AO) extracted data from all included studies, and a second independent reviewer (JR, NE, and LN) each extracted relevant data from a third of the included studies. Discrepancies observed from the independently extracted data of each study were resolved in discussion (*n* = 2).

### 2.5 Quality assessment

The Critical Appraisal Skills Programme checklist (Singh, 2013) was used to assess the quality of the qualitative studies included in the review, and the National Institutes of Health Quality Assessment Tool for Observational Cohort and Cross‐Sectional Studies (National Heart Lung and Blood Institute, 2014) was used to assess the quality of the included quantitative studies. The quality of each study was rated as good, fair, or poor. All included studies were assessed for quality by two reviewers independently: AO assessed the quality for all included studies, and three other reviewers (JR, NE, and LN) divided the included studies between themselves and each assessed a portion of the included studies for quality.

### 2.6 Data synthesis

An integrated mixed‐methods approach with elements of framework synthesis was used to synthesise the findings from individual studies. An integrated mixed‐methods approach combines both quantitative and qualitative data into a single synthesis by either converting qualitative data into numerical format and included in statistical analysis or converting quantitative data into themes that can be coded and presented with qualitative data (The Joanna Briggs Institute, 2014). An *a priori* framework was identified, providing a pre‐existing structure for the organisation and analysis of data, which is referred to as the framework synthesis approach (Barnett‐Page & Thomas, 2009). The *a priori* framework was informed from existing literature and included themes from the existing literature, and additional themes that emerged from the review data were included during the analysis process as shown in Table 1. The data synthesis involved the following stages: familiarisation with the data, coding, identifying a thematic framework, charting the data into the framework matrix, and interpretation and was completed by one researcher with the guidance of an experienced qualitative researcher.

**Table 1:** Framework development for qualitative data analysis

*A priori* framework	Data‐driven factor	Adapted framework for data analysis
Breastfeeding practices • breastfeeding initiation • breastfeeding duration • complementary feeding (introduction of solids) ○ type of solid introduced	Breastfeeding practices • breastfeeding initiation • breastfeeding duration (by modes of breastfeeding) • breastfeeding duration (total) • complementary feeding ○ type of solids introduced ○ time to introduce solids • strategies to encourage breastfeeding	Breastfeeding practices • breastfeeding initiation • breastfeeding duration (by modes of breastfeeding) • breastfeeding duration (total) • complementary feeding (introduction of solids) ○ type of solids introduced ○ time to introduce solids • strategies to encourage breastfeeding
Beliefs, attitudes, and knowledge of breastfeeding • knowledge ○ benefits ○ lack of knowledge • beliefs ○ reasons for choosing breastfeeding/how belief influences choice ○ milk sufficiency • attitudes ○ breastfeeding in public ○ breastfeeding and physical appearance ○ breastfeeding and attachment	Beliefs, attitudes, and knowledge of breastfeeding • knowledge of ○ benefits of breastfeeding ○ exclusive breastfeeding • beliefs ○ reasons for choosing breastfeeding ○ optimal duration of breastfeeding ○ milk sufficiency ○ colostrum ○ water • attitudes ○ breastfeeding in public ○ the use of breast pumps ○ advice and information	Beliefs, attitudes, and knowledge of breastfeeding • knowledge ○ exclusive breastfeeding ○ benefits of breastfeeding • beliefs ○ reasons for choosing breastfeeding ○ optimal duration of breastfeeding ○ milk sufficiency ○ colostrum ○ water • attitudes ○ breastfeeding in public ○ the use of breast pumps ○ advice and information
Influence of socio‐demographics, economic, and cultural factors on infant feeding • socio‐demographics ○ income and finance ○ education level ○ employment ○ acculturation • cultural factors ○ traditional beliefs ▪ special diets and health	Influence of socio‐demographics, economic, and cultural factors on infant feeding • socio‐demographics ○ migration and acculturation ○ employment status ○ family demands • cultural factors ○ desire for big baby • economic factors ○ income status	Influence of socio‐demographics, economic, and cultural factors on infant feeding • socio‐demographics ○ migration and acculturation ○ employment status ○ family demands • cultural factors ○ desire for big baby • economic factors ○ income status
Family support and influence of husbands and grandparents on infant‐feeding method • husbands influence • grandparents influence • mothers' responses to support	Family support • support from husbands • support from mothers (infants' grandmother) • support from other female friends and family • mothers' responses to these support	Support system • support from friends and family ○ grandmothers ○ husbands ○ other female friends and family • support from health professionals • mothers' responses to support
Support from health professionals	Support from health professionals	
Not applicable	Perception of health professionals about the breastfeeding experiences of African immigrant mothers	Perception of health professionals about the breastfeeding experiences of African immigrant mothers.

*Note.* Themes were adapted from existing *a priori* framework and emergent data from studies included in the review and developed into a final framework used in the qualitative data synthesis. Themes that were present in the a priori framework and absent from the studies included in the review were not included in the final framework used in the synthesis.

## 3 RESULTS

**Figure 1:**
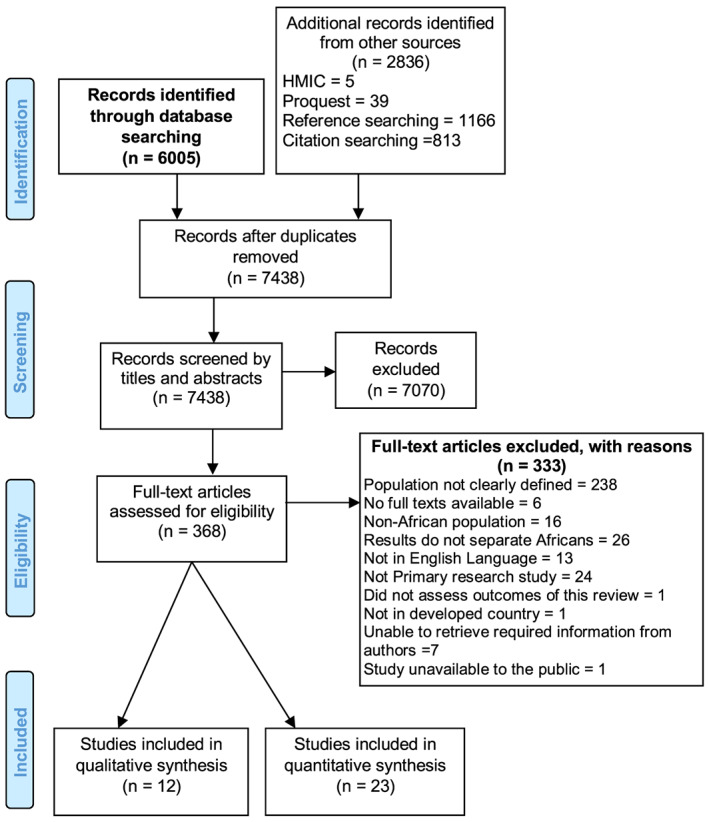
Preferred Reporting Items for Systematic Review and Meta‐Analysis flow diagram showing process of study selection. HMIC, Health Management Information Consortium

A total of 6,005 studies were identified from database searching, and an additional 2,836 studies were retrieved from reference and citation searching, hand‐searching of relevant journals, and grey literature. After removing duplicates and screening by titles and abstracts, 368 full text studies were assessed against the inclusion and exclusion criteria. Thirty‐five studies met all inclusion criteria (Figure 1).

### 3.1 Description of included studies

Twelve of the included studies were qualitative (Castaldo, Mirisola, Costanzo, & Marrone, 2017; Fabiyi, Peacock, Hebert‐Beirne, & Handler, 2016; Gallegos et al., 2015; Hill, Hunt, & Hyrkäs, 2012; Hufton & Raven, 2016; Ingram, Cann, Peacock, & Potter, 2008; Kolanen, Valimaki, & Vehvilainen‐Julkunen, 2016; Steinman et al., 2010; Textor et al., 2013; Twamley et al., 2011; Tyler et al., 2014; Wandel et al., 2016), and 23 were quantitative studies (Brick & Nolan, 2014; Bulk‐Bunschoten, Pasker‐de Jong, van Wouwe, & de Groot, 2008; Busck‐Rasmussen, Villadsen, Norsker, Mortensen, & Andersen, 2014; de Hoog, van Eijsden, Stronks, Gemke, & Vrijkotte, 2011; Dennis et al., 2014; Farchi, Asole, Chapin, & Di Lallo, 2016; Fawzi et al., 1997; Goel, House, & Shanks, 1978; Grewal, Andersen, Sellen, Mosdol, & Torheim, 2016; Griffiths, Tate, & Dezateux, 2005, 2007; Jones & Belsey, 1977; Kelly, Watt, & Nazroo, 2006; Meftuh, Tapsoba, & Lamounier, 1991; Merewood et al., 2007; Moore, Nanthagopan, Hammond, Milligan, & Goff, 2013; Neault et al., 2007; Nolan & Layte, 2015; Parker et al., 2017; Rio et al., 2011; Rubin, Inbar, & Rishpon, 2010; Treuherz, Cullinan, & Saunders, 1982; Wallby & Hjern, 2009). Ten studies were conducted in the United Kingdom (Goel et al., 1978; Griffiths et al., 2005, 2007; Hufton & Raven, 2016; Ingram et al., 2008; Jones & Belsey, 1977; Kelly et al., 2006; Moore et al., 2013; Treuherz et al., 1982; Twamley et al., 2011); eight in the United States (Fabiyi et al., 2016; Hill et al., 2012; Meftuh et al., 1991; Merewood et al., 2007; Neault et al., 2007; Parker et al., 2017; Steinman et al., 2010; Textor et al., 2013); three in Australia (Gallegos et al., 2015; Tyler et al., 2014); two each in Norway (Grewal et al., 2016; Wandel et al., 2016), The Netherlands (Bulk‐Bunschoten et al., 2008; de Hoog et al., 2011), Italy (Castaldo et al., 2017; Farchi et al., 2016), Ireland (Brick & Nolan, 2014; Nolan & Layte, 2015), and Israel (Fawzi et al., 1997; Rubin et al., 2010); and one each in Sweden (Wallby & Hjern, 2009), Spain (Rio et al., 2011), Canada (Dennis, et al., 2014), Denmark (Busck‐Rasmussen et al., 2014), and Finland (Kolanen et al., 2016). A summary of the key characteristics and quality rating of the included studies is presented in Table 2.

**Table 2:** Characteristics of included studies

Author and year	Study location and year of data collection	Sample	Study design	Outcomes measured/explored	Quality assessment summary
Brick & Nolan, 2014	Republic of Ireland 2004–2010	230,750 healthy term babies (number of Africans and country of origin not specified)	Cohort	Determinants of breastfeeding at hospital discharge with a particular focus on maternal country of birth, and the extent to which this is due to maternal characteristics	Good
Bulk‐Bunschoten et al., 2008	Netherlands 1998	4,438 mothers (135 Moroccans).	Survey	Reasons for discontinuing breastfeeding.	Fair, sampling strategy not clearly defined.
Busck‐Rasmussen et al., 2014	Denmark 2002–2009	42,420 children‐mother pair (292 Moroccans).	Cohort	Full breastfeeding until 4 months of age, suboptimal breastfeeding.	Fair, sampling strategy not clearly defined.
Castaldo et al., 2017	Italy 2013 2014	Mothers of 46 Asian and African immigrant children and adolescents (23 Africans—Country not specified).	In‐depth semi‐structured face‐to‐face interviews	Barriers to breastfeeding, the effects of breastfeeding on the psychological and physical health of infants, the social and domestic consequences that affect those women who did not stop breastfeeding when they felt they should have.	Fair, no clear statement of aim.
de Hoog et al., 2011	Amsterdam 2003–2004	3,702 mother–child pairs (282 Moroccans).	Cohort	Change in standard deviation scores (ΔSDS) for weight, length, and weight‐for‐length.	Fair, sampling strategy not clearly defined.
Dennis, Gagnon, Van Hulst, & Dougherty, 2014	Canada 2006–2009	1,875 immigrants and Canadian born women (169 Africans—Country not specified).	Prospective cohort	Predictors of exclusive breastfeeding at 16 weeks post‐partum.	Fair, sampling strategy not clearly defined.
Fabiyi et al., 2016	Ohio, US 2012–2013	20 Black mothers (10 African‐born—Country not specified)	Semi‐structured interviews	Mothers' experiences and views about infant feeding (bottle‐feeding and breastfeeding) while growing up, during pregnancy, and since the delivery of the infant; the role that family members, friends, and health providers played in those experiences; and the barriers and challenges that participants encountered during infant feeding in the most recent pregnancy.	Good
Farchi et al., 2016	Lazio, Italy 2006–2011	6,505 mothers with healthy newborns (111 Africans—Country not specified)	Cohort	Breastfeeding during hospital stay.	Fair, no adjustments for confounding variables.
Fawzi et al., 1997	Israel 1982–1986	1,040 pregnant women of African descent—Country not specified.	Cohort	Maternal anthropometry, infant feeding practices at 1, 2, 3, and 6 months and infant anthropometry.	Fair, outcome measures not clearly defined and no adjustment for confounding variables.
Gallegos et al., 2015	Brisbane and Perth, Australia 2007–2008	30 women and 1 man; 3 women born in Sierra Leone; 8 women and 1 man born in Liberia; 4 women born in Burundi; and 15 women born in Congo.	Face‐to‐face interviews and focus groups	Cultural beliefs, traditional practices, barriers and enablers, and personal experiences in both the country of origin and Australia regarding breastfeeding.	Good
Goel et al., 1978	Glasgow, UK 1974–1976	506 children (99 Africans—Country not specified).	Cross‐sectional survey	Type of feeding mode used, relation of country of birth to feeding mode, duration of breastfeeding, time of introduction of solids, type of solids given, and vitamin supplements.	Fair, no adjustments for confounding variables and sampling strategy not clearly defined.
Grewal et al., 2016	Eastern Norway (Oslo, Akershus and Buskerud) 2013–2014	187 participants (107 of Somali origin).	Cross‐sectional survey. Retrospective	Exclusive breastfeeding, breastfeeding, and other complementary feeding practices at 6 months of age and retrospectively from birth.	Good
Griffiths et al., 2005	UK 2000–2002	18,150 natural mothers of singleton infants. (358 Black Africans—Country not specified).	Cohort	Breastfeeding initiation, measures of breastfeeding duration and prevalence as any breastfeeding to: At least 1 month (>4.35 weeks); 4 months (>17.4 weeks); and 6 months (>26.1 weeks) of age.	Fair, sampling strategy not clearly defined.
Griffiths et al., 2007	UK 2000–2002	18,150 natural mothers of singleton infants. (358 Black Africans—Country not specified).	Cohort	Breastfeeding initiation, breastfeeding discontinuation, and introduction of solid foods before 4 months.	Fair, sampling strategy not clearly defined.
Hill et al., 2012	North‐eastern US	18 Somali women.	Focus groups	Health care experiences and beliefs regarding pregnancy and birth in the Unites States	Good
Hufton & Raven, 2016	Liverpool and Manchester, UK 2012	30 refugees (24 Africans—Country not specified).	Semi‐structured interviews and focus groups	U.K. feeding experiences compared with experiences elsewhere, knowledge, and awareness of U.K. feeding recommendations, difficulties encountered with infant feeding methods and where help is sought.	Fair, no clear description of analysis process.
Ingram et al., 2008	Bristol, UK 2006–2007	22 women (5 Somali).	Focus groups	Barriers to exclusive breastfeeding to 6 months.	Fair, no clear description of analysis process.
Jones & Belsey, 1977	Lambeth, London, UK 1975	280 mothers of 12‐week‐old infants (14 Africans—Country not specified)	Cross‐sectional survey	Factors influencing mothers' choice of infant feeding.	Good
Kelly et al., 2006	UK 2000 2001	321 Black African mothers—Country not specified.	Survey involving face‐to‐face interviews	Breastfeeding (exclusive, predominant or any) rates in first 6 months	Fair, sampling strategy not clearly defined
Kolanen et al., 2016	Finland 2012	7 Somali mothers.	Focus groups with semi‐structured questions	Breastfeeding in the Somali culture.	Poor, no clear description of research design, recruitment strategy and data analysis process.
Meftuh et al., 1991	Los Angeles and San Diego, United States 1987	45 Ethiopian mothers.	Retrospective In‐depth interview	Prenatal experiences and infant feeding patterns.	Good
Merewood et al., 2007	Boston, US 2003	336 singleton infants (32 Africans including Cape Verde).	Cross‐sectional	Breastfeeding initiation, breastfeeding duration, and factors associated with continued breastfeeding.	Good
Moore et al., 2013	London, UK 2010–2011	349 BME (107 Black Africans—Country not specified).	Survey	Weaning behaviours—Weaning age, factors associated with weaning decisions, weaning information sources, engagement with medical advice, and etc.	Fair, no adjustments for confounding variables
Neault et al., 2007	US 1998–2004	8,800 children aged 0–3 years (1,078 Africans—Country not specified)	Cohort	Infant health status, history of chronic illness, hospitalisation history, and growth status.	Fair, sampling strategy not clearly defined
Nolan & Layte, 2015	Ireland 2007–2009	9,700 9‐month‐old children (African = 1.5%); 7,200 9‐year‐old children (African = 1.3%)—Countries not specified.	Cohort	Breastfeeding initiation.	Good
Parker et al., 2017	US 2011–2014	3983 mothers enrolled African‐born = 42	Cohort	Safe sleep and breastfeeding practices.	Good
Rio et al., 2011	Catalonia and Valencia, Spain 2005–2006	2105 sub‐Saharan Africans—Country not specified.	Cross‐sectional	Breastfeeding initiation	Fair, sampling strategy not clearly defined
Rubin et al., 2010	Hadera, Israel 2005–2006	93 Ethiopian born mothers	Cross‐sectional study	Association between the duration of breastfeeding and the independent variables (marital status, educational level, number of children, employment status, time from date of immigration, and religious observance)	Fair, sampling strategy not clearly defined and no adjustments for confounding variables
Steinman et al., 2010	Seattle, US (year not stated)	37 Somali mothers	Focus groups	Beliefs about infant feeding, hunger and ideal weight, feeding practices, nutrition education approaches, and provider/mother interactions.	Good
Textor et al., 2013	South‐eastern Minnesota, US 2010–2011	9 immigrant mothers (5 Somali)	Semi‐structured interviews (mothers) and focus groups (nurses)	Breastfeeding experiences, attitudes and practices related to breastfeeding, and perceptions of relationships with health care providers.	Poor, no clear description of research design, recruitment strategy and data analysis process.
Treuherz et al., 1982	The City and East London districts, UK 1979–1980	3,712 babies 4 weeks of age (191 Africans—Country not specified).	Prospective cohort	Type of feeding (breastfeeding, bottle feeding or mixed)	Fair, sampling strategy not clearly defined
Twamley et al., 2011	London and Birmingham, UK	34 ethnic minority women born in the United Kingdom (2 Africans—Country not specified).	Semi‐structured interviews	Pregnancy, birth, caring for the newborn, infant feeding, and family and partner involvement in decisions around care.	Fair, no clear description of data analysis process.
Tyler et al., 2014	Toowoomba, Australia. (year not stated)	10 Sudanese women	Semi‐structured interviews	Commonalities and differences in the Sudanese mothers' breastfeeding experiences in Africa and Australia.	Poor, no clear description of recruitment strategy and data analysis process.
(Wallby & Hjern, 2009)	Uppsala, Sweden 1997–2001	12,197 infants (212 Africans—Country not specified).	Cohort	Breastfeeding at 1 week, 6, and 12 months	Good
(Wandel et al., 2016)	Oslo, Norway 2012–2015	21 Somali mothers.	Semi‐structured interview and focus groups	Mothers' experiences with breastfeeding and complementary feeding, and the introduction of family food.	Good

*Note.* Quality assessment rating description: A study was rated “good” if the risk of bias was considered minimal, “fair” if there was some risk of bias but not sufficient to make the results invalid, and “poor” if there was substantial risk of bias that could significantly affect the interpretation of the results.

Abbreviation: BME, Black and minority ethnicity; NHB, Non‐Hispanic black.

The majority of the participants under study were immigrant mothers from Somalia (Grewal et al., 2016; Hill et al., 2012; Ingram et al., 2008; Kolanen et al., 2016; Steinman et al., 2010; Textor et al., 2013; Wandel et al., 2016), Ethiopia (Meftuh et al., 1991; Rubin et al., 2010), and Morocco (Bulk‐Bunschoten et al., 2008; Busck‐Rasmussen et al., 2014; de Hoog et al., 2011). One study included African mothers from Burundi, Congo, Liberia, and Sierra Leone (Gallegos et al., 2015), whereas other studies (Brick & Nolan, 2014; Castaldo et al., 2017; Dennis et al., 2014; Fabiyi et al., 2016; Farchi et al., 2016; Fawzi et al., 1997; Goel et al., 1978; Griffiths et al., 2005, 2007; Hufton & Raven, 2016; Jones & Belsey, 1977; Kelly et al., 2006; Merewood et al., 2007; Moore et al., 2013; Neault et al., 2007; Nolan & Layte, 2015; Parker et al., 2017; Rio et al., 2011; Treuherz et al., 1982; Twamley et al., 2011; Wallby & Hjern, 2009) described the study population as Africans or Black Africans, without specifying which African country the mothers included in the study originated from.

### 3.2 Breastfeeding practices

Twenty‐eight studies reported on breastfeeding practices (Brick & Nolan, 2014; Bulk‐Bunschoten et al., 2008; Busck‐Rasmussen et al., 2014; Castaldo et al., 2017; de Hoog et al., 2011; Fabiyi et al., 2016; Farchi et al., 2016; Fawzi et al., 1997; Gallegos et al., 2015; Goel et al., 1978; Grewal et al., 2016; Griffiths et al., 2005, 2007; Hufton & Raven, 2016; Ingram et al., 2008; Jones & Belsey, 1977; Kelly et al., 2006; Kolanen et al., 2016; Meftuh et al., 1991; Merewood et al., 2007; Moore et al., 2013; Neault et al., 2007; Nolan & Layte, 2015; Parker et al., 2017; Rio et al., 2011; Steinman et al., 2010; Twamley et al., 2011; Wandel et al., 2016). Due to the variability in reporting across studies, it was not possible to pool quantitative data into a meta‐analysis.

#### 3.2.1 Breastfeeding initiation

Breastfeeding initiation rates are presented in Table 3. The average initiation rate across studies calculated using the corresponding sample sizes from each study was 90.2% for the 4,345 participants across all of the included studies. One study reported the odds of initiating breastfeeding of African as compared with “White” mothers to be 10 times more after adjusting for covariates (Kelly et al., 2006). Timing for initiation of breastfeeding was reported in one study as 93% initiation within 24 hr (Grewal et al., 2016).

**Table 3:** Breastfeeding initiation rates

Study reference	Number of participants	BF initiation (%)
Castaldo et al., 2017	23	100
de Hoog et al., 2011	232	92.2
Fabiyi et al., 2016	20	100
Grewal et al., 2016	107	93^a^
Griffiths et al., 2005	358	95
Hufton and Raven, 2016	13^b^	100
Jones & Belsey, 1977	14	86
Kolanen et al., 2016	11	100
Meftuh et al., 1991	45	100
Merewood et al., 2007	32	100
Neault et al., 2007	1078	88
Nolan et al, 2015^c^	240	84^d^ 84.3^e^
Parker et al., 2017	42	96.9
Rio et al., 2011	2105	90
Twamley et al., 2011	2	100
Wandel et al., 2016	22	100
Kelly et al., 2006, f	321	8.1 (4.4–14.7)^g^13.6 (7.8–23.7)^h^ 10.5(6.1–18.2)^i^

Abbreviation: BF, breastfeeding.

a Within 24 hr.

b Only human immunodeficiency virus‐negative mothers reported and one mother was still pregnant.

c Two cohorts studied.

d Cohort 1 (C1) = an infant cohort of 9‐month‐old children.

e Cohort 2 (C2) = a chid cohort of 9‐year‐old children.

f Not included in aggregate percentage calculation.

g Crude odds ratio (OR).

h OR adjusted for gender of baby, parity, age of mother, housing tenure, household income, mother's education, mother's occupational social class, smoking status, mother's employment status, one or two parent household, and child care arrangements,

i OR further adjusted for language spoken at home.

#### 3.2.2 Breastfeeding duration

Breastfeeding rates between 1 week and 6 months are presented in Table 4. Studies describing breastfeeding rates at hospital discharge were reported as breastfeeding in the first week of life. Breastfeeding rates at 5 and 6 months of age were presented together. Total duration of exclusive breastfeeding or any breastfeeding is presented in Table 5 and ranged from 12 weeks (Bulk‐Bunschoten et al., 2008) to 3 years (Goel et al., 1978).

**Table 4:** Breastfeeding rates in percentages according to type of feeding practices between 1 week and 6 months after birth

Study reference	No.	BF at hospital discharge or week 1 (%)					Breastfeeding at 1 month (%)					Breastfeeding at 2 months (%)					Breastfeeding at 3 months (%)					Breastfeeding at 4 months (16–17 weeks) (%)					Breastfeeding at 5–6 months (%)				
		EBF	PBF	MF	Any BF	No BF	EBF	PBF	MF	Any BF	No BF	EBF	PBF	MF	Any BF	No BF	EBF	PBF	MF	Any BF	No BF	EBF	PBF	MF	Any BF	No BF	EBF	PBF	MF	Any BF	No BF
Brick & Nolan, 2014					83.8^a^ 0.45 (0.025)^b^																										
Bulk‐Bunschoten et al., 2008	135	80.0			90.0																	<20.0			<40.0						
Busck‐Rasmussen et al., 2014	292																					49.2									
Castaldo et al., 2017	23																			61.0										43.0	
De Hoog et al, 2011	232				5.6	7.8					11.9										37.8	32.7									58.1
Farchi et al., 2016	111	70.3	3.60	26.1		0.0																									
Fawzi et al., 1997	351								30.0	34.0	36.0			21.0	18.0	62.0			17.0	6.0	77.0									0.2	
Goel et al., 1978	99																													79.2	20.8
Grewal et al., 2016	107						37.0		44.0	97.0							21.0					7.0		67.0			0			79.0	
Griffiths et al., 2007	334																								38						
Hufton et al, 2016	13^c^	38.5	7.7	30.8		15.4											38.5	7.7	30.8		15.4	38.5	0	30.8		23.1	30.8	0	38.5		23.1
Ingram et al., 2008	5																					60.0		40.0							
Kelly et al., 2006	321																	24.0		5.3 (3.3–8.7)^d^ 7.4 (4.2–13.2)^e^ 6.0 (3.3–10.8)^f^											
Kolanen et al., 2016	11	9.10		90.9																							9.0		72.7		18.2
Merewood et al., 2007	32																													72.7	27.3
Parker et al., 2017	42											35.2																			
Rubin et al., 2010	93																					76.3	18.3			5.4					
Treuherz et al., 1982	191								48.7	33.0	18.3																				
Twamley et al., 2011	2	50.0		50.0																										50.0	

Abbreviations: BF, breastfeeding; EBF, exclusive breastfeeding; MF, mixed feeding; PBF, predominant breastfeeding.

a Average percentage over 7 years.

b Marginal effect (standard Error) of breastfeeding if African compared with Irish‐born mothers.

c Only human immunodeficiency virus‐negative mothers reported and one mother was still pregnant.

d Crude odds ratio (OR) of breastfeeding if African compared with “White” mothers.

e OR adjusted for gender of baby, parity, age of mother, housing tenure, household income, mother's education, mother's occupational social class, smoking status, mother's employment status, one or two parent household, and child care arrangements.

f OR further adjusted for language spoken at home.

**Table 5:** Duration of breastfeeding

Study reference	No. of participants	BF duration	
		EBF	Any BF
Bulk‐Bunschoten et al., 2008	135	3 weeks^a^	12 weeks^a^
Busck‐Rasmussen et al., 2014	292	Risk of suboptimal BF (EBF for <4 months) = 1.67 (1.26–2.22)	NR
Castaldo et al., 2017	23	NR	Between 1 month and 2 years: 17% (3–6 m), 13% (6‐12 m), 31% (1‐2y)
Fabiyi et al., 2016	20	NR	9–12 months
Goel et al., 1978	99	NR	> 1 year, up to 3 years = 5%
Grewal et al., 2016	107	NR	20.6% stopped before 6 months
Griffiths et al., 2007	334	NR	0.6 (0.5–0.7)^d^ 0.7(0.6–0.8)^e^
Kolanen et al., 2016	11	NR	7.8 months^b^
Meftuh et al., 1991	45	NR	4.2 months^b^
Moore et al., 2013	107	NR	31% weaned at 17 weeks
Rubin et al., 2010	93	NR	19.7 ± 12.4 months^c^
Steinman et al., 2010	37	NR	Approximately 1 year

Abbreviations: BF, breastfeeding; EBF, exclusive breastfeeding; NR, not reported.

a Median.

b Mean.

c Mean ± standard deviation.

d Crude rate ratio of BF cessation before 4 months (African vs. “White” mothers).

e Adjusted rate ratio of BF cessation before 4 months (African vs. White mothers).

#### 3.2.3 Complementary feeding

The age at which mothers introduced solids to their infants ranged from 3 (Gallegos et al., 2015) to 6 months or more (de Hoog et al., 2011; Moore et al., 2013; Steinman et al., 2010). Complementary foods given also varied widely but was mostly ready‐made shop‐bought baby food (Gallegos et al., 2015; Grewal et al., 2016), baby cereal/rice/pasta (Steinman et al., 2010), fruits (Grewal et al., 2016; Steinman et al., 2010), homemade porridge, or other cereals (Grewal et al., 2016).

#### 3.2.4 Strategies to encourage breastfeeding

Among Somali mothers, ways to recognise infants' hunger and satiety were discussed. These included the infant's body language, ability of the infant to sleep and timing of infants' feeding, with timing of infant feeding being the most common method adopted (Steinman et al., 2010). Mothers who did not adhere to specified timings to feed their infants expressed difficulty in understanding infant's feeding needs or hunger status (Bulk‐Bunschoten et al., 2008; Steinman et al., 2010). This contributed to early cessation of breastfeeding among Moroccan mothers in one study (Bulk‐Bunschoten et al., 2008). Other infant‐related reasons leading to early cessation of breastfeeding were colic, constipation, and vomiting (Bulk‐Bunschoten et al., 2008). Mothers in the included studies further highlighted strategies they employed to increase their milk supply during lactation, such as the consumption of certain foods (Gallegos et al., 2015; Steinman et al., 2010), having a healthy appetite (Textor et al., 2013), breastfeeding more (Steinman et al., 2010), increasing fluid intake (Steinman et al., 2010), or simply focusing on the child's needs (Kolanen et al., 2016). In some cases, however, the mothers were not certain how milk supply could be increased and suggested that it may be dependent on an individual's make‐up and cannot be modified (Steinman et al., 2010).

### 3.3 Knowledge, beliefs, and attitudes towards breastfeeding

#### 3.3.1 Knowledge of breastfeeding

Three studies (Fabiyi et al., 2016; Hufton & Raven, 2016; Twamley et al., 2011) describing participants as Africans reported on the mothers' awareness of the health, nutritional, and emotional benefits of breastfeeding, referring to the “antibodies transferred to the infant” and “reduced chances of breast cancer for the mother” in one study (Twamley et al., 2011), “strong bones,” “strength,” and “good immunity” in another (Hufton & Raven, 2016), being “easier on the baby's digestive system” and “prevent illness” in another (Fabiyi et al., 2016). The “economic benefit of not having to purchase formula”, “the emotional connection with the infant”, “weight loss of mother”, “the convenience of having a ‘supply ready to go’”, and “the developmental benefits to the infant” were other benefits highlighted in one study (Fabiyi et al., 2016). Knowledge about EBF was not reported in most studies, but one study (Wandel et al., 2016) showed that Somali mothers expressed uncertainty about what EBF meant and what it entailed.

#### 3.3.2 Beliefs around breastfeeding

The mothers described breastfeeding as “the natural thing to do” (Gallegos et al., 2015; Wandel et al., 2016), “the norm” (Gallegos et al., 2015; Hufton & Raven, 2016; Textor et al., 2013), “a better source of nutrition” (Gallegos et al., 2015); and a “typical” thing to pass on from one generation to the next (Textor et al., 2013). One study (Fabiyi et al., 2016) of African‐born versus African‐American mothers reported African‐born women recalling more memories of breastfeeding during their childhood and spoke of how widely practiced and accepted it was in their home countries.

The main reasons for choosing breastfeeding among the mothers were the health benefit to the infant, such as providing immunity and strength to infants (Hufton & Raven, 2016), aiding infants' development (Fabiyi et al., 2016) and its medicinal benefits (Kolanen et al., 2016), and religion (Hufton & Raven, 2016;Kolanen et al., 2016 ; Steinman et al., 2010 ; Wandel et al., 2016). The mothers, particularly those of Islamic religion, agreed that the optimal duration of breastfeeding should be between 2 and 2.5 years (Gallegos et al., 2015; Hufton & Raven, 2016; Steinman et al., 2010; Wandel et al., 2016) as instructed in the Qur'an. Other reasons for breastfeeding were presented in one study and included mother–infant closeness, infant's preference, soothing crying baby, and putting baby to sleep (Steinman et al., 2010).

Beliefs around colostrum varied across studies. Two studies (Steinman et al., 2010; Textor et al., 2013) reported that Somali mothers believed in the tradition that the colostrum is “dirty milk” or *danbar* in Somali language because it has stayed too long in the breasts (Textor et al., 2013) and could make a baby sick (Steinman et al., 2010). On the contrary, Somali mothers in another study considered colostrum as being fresh because it is the first milk the mother produces (Steinman et al., 2010). Notwithstanding, these mothers held strongly to the belief that milk that sits in the breasts longer than 2 hr is old (Steinman et al., 2010). Some of the Somali mothers had only given colostrum to their infants after migration and being informed by health professionals of its benefits (Steinman et al., 2010). Among other African mothers from Sierra Leone, Congo, Burundi and Liberia, colostrum was deemed useful in cleansing the infant's intestines (Gallegos et al., 2015).

The mothers held strong opinions about giving an infant water within the first week of life. Some believed it was necessary because breastfeeding makes the infant thirsty (Wandel et al., 2016), others used it for cleansing the infant's intestines (Gallegos et al., 2015), whereas others reported giving sweetened water, containing either sugar or honey at breastfeeding initiation (Kolanen et al., 2016). Some mothers reported that they only gave water in the summer when the weather was warmer, believing that the infant will need the extra fluid (Wandel et al., 2016).

#### 3.3.3 Attitudes towards breastfeeding

A study on Somali mothers reported that mothers “displayed great affection” towards breastfeeding (Gallegos et al., 2015), and another study stated that the mothers “like breastfeeding and consider themselves good at it” (Hill et al., 2012). One study (Gallegos et al., 2015) indicated that Somali mothers often practice sexual abstinence during the first year of an infant's life to encourage breastfeeding.

Attitudes towards the use of breast pumps showed that the mothers were not familiar with breast pumps and its use (Hill et al., 2012;Steinman et al., 2010 ; Wandel et al., 2016). They described breast pumps as being too cumbersome (Wandel et al., 2016), difficult, and not a viable option if they were to breastfeed for as long as 2 years (Hill et al., 2012), as was the usual practice of the mothers. Some mothers expressed interest in using breast pumps to continue breastfeeding for longer by saving up milk and increasing supply, but they either had limited experience with its use or were sceptical due to the belief that “breast milk spoils after too much time in the breasts” (Steinman et al., 2010). One study, however, reported that some African mothers had fed expressed milk to their infants (Fabiyi et al., 2016).

With respect to breastfeeding in public, the mothers stated that some stigma and shame were associated with breastfeeding in public in their host countries, resulting in the mothers feeding formula milk to their infants when in public (Gallegos et al., 2015; Steinman et al., 2010; Textor et al., 2013; Twamley et al., 2011; Wandel et al., 2016). Factors such as the lack of visibility of public breastfeeding (Gallegos et al., 2015), traditional unacceptability (Steinman et al., 2010; Twamley et al., 2011), being Black (Gallegos et al., 2015), and their religion (Islam) (Kolanen et al., 2016; Wandel et al., 2016) were highlighted as reasons they felt “ashamed” to breastfeed in public. Mothers who felt comfortable breastfeeding in public appeared to be mostly Muslims, who stated that their clothing helped them in such situations (Ingram et al., 2008). Notwithstanding, they sought private places such as changing rooms and toilets, preferring places where they would not be seen by men, particularly because they felt the men of their host countries got offended seeing a breastfeeding lady (Gallegos et al., 2015).

Other negative emotions regarding breastfeeding in the HICs were reported. Mothers highlighted feelings of vulnerability and discrimination when interacting with health professionals, due to their minority status (Wandel et al., 2016). They also expressed a fear of fitting in and doing things right (Wandel et al., 2016) and fear and concerns around lactation, work, and their health (Fabiyi et al., 2016). One study (Castaldo et al., 2017) highlighted that some mothers felt sadness, anger, and fear in relation to psychological violence within their families, as well as traumas following religious persecution in their native context, which led them to stop breastfeeding.

### 3.4 Influence of socio‐demographic, economic, and cultural factors

#### 3.4.1 Socio‐demographic factors

The mothers reported a higher likelihood to practice EBF for 6 months in their home countries than to supplement (Ingram et al., 2008; Kolanen et al., 2016) and described the use of formula as “a need to adopt the western approach” (Gallegos et al., 2015). Although one study (Gallegos et al., 2015) related the EBF for 6 months in their home country to a lack of finance to purchase required supplements, or the availability of adequate family and friends support to make the breastfeeding process easier and more effective, five studies (Gallegos et al., 2015; Ingram et al., 2008;Kolanen et al., 2016 ; Steinman et al., 2010 ; Wandel et al., 2016) attributed supplementing with formula milk to being in a Western society. Not having enough milk to meet the baby's demands was seen as a problem associated with lifestyle changes and increased stress levels in HICs (Kolanen et al., 2016; Steinman et al., 2010; Wandel et al., 2016). Although some mothers perceived the need to supplement as “copying the Western culture” (Gallegos et al., 2015), others saw it as being more convenient to balance their lifestyle demands with the care of their infants (Kolanen et al., 2016; Steinman et al., 2010; Wandel et al., 2016), as a way to involve fathers and other family members (Twamley et al., 2011), as a means to achieve a big “healthy” baby that is the acceptable standard within their communities (Gallegos et al., 2015; Ingram et al., 2008; Steinman et al., 2010), or as the result of having insufficient knowledge of the strategies to overcome breast refusal (Steinman et al., 2010).

Additionally, competing demands such as returning to work (Bulk‐Bunschoten et al., 2008; Castaldo et al., 2017; Fabiyi et al., 2016; Hill et al., 2012; Steinman et al., 2010), other children or becoming pregnant again (Castaldo et al., 2017;Kolanen et al., 2016 ; Steinman et al., 2010), housekeeping (Kolanen et al., 2016; Steinman et al., 2010), and other family members (Steinman et al., 2010) were highlighted as factors that encouraged early supplementation with formula in order to receive assistance with feeding the infant from others, or in some cases, stop breastfeeding altogether. The mothers believed that returning to work led to reduced milk supply from insufficient time to breastfeed (Fabiyi et al., 2016; Steinman et al., 2010). Additionally, the mothers described the exhaustion they felt after childbirth (Kolanen et al., 2016; Textor et al., 2013), such that the nurses sometimes had to help with feeding their infants (Textor et al., 2013). This contributed to the decision to stop breastfeeding among some mothers (Bulk‐Bunschoten et al., 2008). Others indicated that in the absence of practical support in HICs, it was difficult to combine breastfeeding with everyday life (Wandel et al., 2016), work, and necessary personal care including having a proper diet and adequate rest (Fabiyi et al., 2016), two factors that were emphasised for achieving adequate milk supply (Kolanen et al., 2016). These demanding situations were said to result in exhaustion, loneliness, and depression but equally served as a source of encouragement for the women (Kolanen et al., 2016).

#### 3.4.2 Cultural factors

One of the major cultural factors affecting breastfeeding practices was the belief that infants need to be big or fat (Bulk‐Bunschoten et al., 2008; Gallegos et al., 2015; Ingram et al., 2008; Steinman et al., 2010; Textor et al., 2013; Twamley et al., 2011; Wandel et al., 2016). The mothers believed that a big or “plump” baby is healthier and has more protection from illnesses, and having the ideal plump was linked to health, strength, and beauty (Gallegos et al., 2015; Steinman et al., 2010; Twamley et al., 2011). The desire to achieve a bigger baby resulted in concerns about the sufficiency of the breast milk they produced for their infants' growth (Castaldo et al., 2017; Gallegos et al., 2015; Kolanen et al., 2016; Steinman et al., 2010; Textor et al., 2013; Wandel et al., 2016) and was one of the reasons for breastfeeding cessation (Bulk‐Bunschoten et al., 2008). Not being able to measure the milk from the breast was considered a challenge, which often led to early supplementation with formula milk (Fabiyi et al., 2016; Steinman et al., 2010; Textor et al., 2013).

The mothers often received praises from older women in their community for doing a good job when their infants were of a certain size (Wandel et al., 2016), described by some as “just the right plump, not over‐fat like obesity, just middle” (Steinman et al., 2010). Two studies (Steinman et al., 2010; Wandel et al., 2016) noted that the desire to have a big baby was strongest during infancy, particularly between 0 and 6 months (Steinman et al., 2010), and reduced as the child approached school age to avoid being teased by peers.

#### 3.4.3 Economic factors

Only one study (Gallegos et al., 2015) discussed on the economic factors influencing breastfeeding. African mothers expressed a sense of increased financial security and financial freedom while living in a HIC, which contributed to early supplementing with formula milk. The mothers expressed that formula milk was more accessible and affordable to them, and they were more likely to use it while in their host country compared with when they were in their home countries.

### 3.5 Support system

#### 3.5.1 Support from friends and family

African mothers reported having a strong support system as a traditional practice in their home countries, particularly from female friends and family members (Fabiyi et al., 2016; Gallegos et al., 2015; Ingram et al., 2008; Kolanen et al., 2016). The most important source of support and information for most of the mothers was their own mothers (infant's grandmother; Gallegos et al., 2015; Hill et al., 2012; Kolanen et al., 2016). A new mother may live with her mother for 1 to 2 years in order to get necessary help with childcare and housekeeping (Gallegos et al., 2015) or have the relative(s) or hired help to help out during the initial 40 days after birth (Kolanen et al., 2016). After migration to a HIC, they noted the absence of this kind of support but highlighted the possibility of replicating such practice by turning to female friends and relations (Gallegos et al., 2015). However, the busy schedules of most individuals in HICs meant that replicating this support system was often challenging and anyone who managed to get some sort of support similar to this was considered “lucky” (Kolanen et al., 2016). The support and information from female family and friends was given more recognition that health professionals' advice was often considered redundant (Kolanen et al., 2016).

Fathers were equally vital in offering support, often in the form of encouragement by recognising breastfeeding as part of the mother's job (Gallegos et al., 2015), and in providing assistance within the household (Kolanen et al., 2016). Traditionally, there was no expectation from African men to get involved with household chores or childcare (Gallegos et al., 2015; Kolanen et al., 2016), but the fathers had increased their involvement in household responsibilities after migration, taking the role of female family and friends in the absence of such support (Kolanen et al., 2016; Twamley et al., 2011).

The kind of support mothers living in HICs received from friends and family included a wide range of information on breastfeeding (Fabiyi et al., 2016; Hill et al., 2012; Ingram et al., 2008; Kolanen et al., 2016; Steinman et al., 2010; Textor et al., 2013) and milk supply (Fabiyi et al., 2016; Hufton & Raven, 2016; Wandel et al., 2016), encouragement to breastfeed (Gallegos et al., 2015; Kolanen et al., 2016), and practical support such as feeding and caring for the infant (Fabiyi et al., 2016; Hufton & Raven, 2016; Ingram et al., 2008; Kolanen et al., 2016; Wandel et al., 2016) and assistance with household chores (Gallegos et al., 2015; Kolanen et al., 2016). Although, mothers were sometimes encouraged by friends and family to offer formula to infants from birth (Ingram et al., 2008; Wandel et al., 2016), infants' grandparents were said to be generally supportive of breastfeeding (Fabiyi et al., 2016; Hufton & Raven, 2016; Ingram et al., 2008; Wandel et al., 2016). However, the mothers felt pressured to listen to and act on breastfeeding advice and information received from friends and family (Fabiyi et al., 2016; Gallegos et al., 2015; Ingram et al., 2008; Textor et al., 2013; Wandel et al., 2016), and feared the stigma and criticism that could result from nonadherence (Gallegos et al., 2015). Whether it was encouraging breastfeeding or supplementing with formula, mothers explained that they lacked the confidence to go against the advice from the women in their community (Ingram et al., 2008). On the other hand, employers were said to be discouraging of breastfeeding (Fabiyi et al., 2016).

#### 3.5.2 Support from health professionals

The mothers highlighted the information and support they received from health professionals in the host country (Hill et al., 2012; Ingram et al., 2008; Kolanen et al., 2016; Steinman et al., 2010; Textor et al., 2013; Twamley et al., 2011; Wandel et al., 2016), which included information on the benefits of breastfeeding, breastfeeding positions, breastfeeding on demand, amount of breast milk needed, skin to skin contact, and rooming in with their babies. Although this information was valued, the information received from health professionals sometimes conflicted their traditional beliefs and information from friends and family (Fabiyi et al., 2016; Ingram et al., 2008; Steinman et al., 2010; Textor et al., 2013; Twamley et al., 2011; Wandel et al., 2016), reducing the value of the Health professionals' advice (Wandel et al., 2016). However, some mothers reported using their own judgements to make decisions between health professionals' advice and their traditional beliefs (Steinman et al., 2010; Wandel et al., 2016), especially women with previous experiences. Visits to the health clinics to obtain health professionals support, particularly in urban areas were sometimes considered stressful and worthless, and other times, they were reported as being very positive (Wandel et al., 2016).

One major barrier identified by the mothers to receiving adequate support in the host environment was their language, as they were not fluent in the language of the host country (Gallegos et al., 2015; Kolanen et al., 2016; Steinman et al., 2010; Wandel et al., 2016) and could not always get an interpreter (Gallegos et al., 2015; Kolanen et al., 2016; Wandel et al., 2016). The absence of an interpreter potentially led to feelings of loneliness (Kolanen et al., 2016). The mothers expressed a desire for additional and more “concrete” information, such as an understanding of “supply and demand” in breastfeeding and breast milk sufficiency to help them understand and deal with the challenges of breastfeeding (Fabiyi et al., 2016; Hufton & Raven, 2016; Ingram et al., 2008; Wandel et al., 2016), preferably in their native languages for easier comprehension (Steinman et al., 2010). Mothers also discussed the desire for additional support in terms of peer support groups (Hufton & Raven, 2016), support from employers and workplaces (Fabiyi et al., 2016), lactation support following discharge from hospital (Fabiyi et al., 2016), as well as support groups set up for women of their culture (Ingram et al., 2008).

### 3.6 Perception of health professionals

Three studies (Hufton & Raven, 2016;Textor et al., 2013 ; Twamley et al., 2011) reported on the perception of health professionals. Health professionals' perceptions were in agreement with the reports of the mothers, stating that African mothers had a tendency to introduce formula to their infants early, mainly due to their cultural beliefs (Textor et al., 2013; Twamley et al., 2011). One such belief was that breast milk is not produced in the initial few days after delivery and that the first breast milk produced (colostrum) was “bad” or “dirty” (Textor et al., 2013), which resulted in delayed initiation of breastfeeding and the use of formula within the first few days of the infants' life (Textor et al., 2013). Another such belief was the desire to have a big baby, which resulted in mix‐feeding or “topping‐up,” an on‐going and common practice among African mothers (Twamley et al., 2011).

The health professionals also acknowledged the influence of family and friends in encouraging supplementation with formula among African mothers, a practice that led to disappointment and frustration among health professionals (Twamley et al., 2011). They explained that their biggest challenge in counselling immigrant mothers about breastfeeding was the cultural differences (Textor et al., 2013). Health professionals reported feeling unprepared to support women with different beliefs because the women sometimes were not interested in what they had to say or did not trust them (Textor et al., 2013) and seemed to value advice from their mothers more than from the nurses. They also reported that the presence of other family members during support sessions with the mothers interfered with their session and the mothers were reluctant to divulge information or breastfeed in such settings, due to their beliefs about exposing body parts (Textor et al., 2013). Health professionals felt that the cultural competence seminars they attended did not provide them with sufficient information and confidence to support immigrant mothers. Some health professionals reported supplying formula to the mothers because they were uncertain on how to alley their concerns (Textor et al., 2013).

Language was also highlighted by health professionals to be a major barrier to supporting immigrant mothers, even with the use of interpreters as they worried whether the interpreters were passing across the right messages. Additionally, most of the interpreters were male, which was considered inappropriate for breastfeeding support, and was therefore counterproductive (Textor et al., 2013). The need for increased awareness at local and national levels, as well as additional support and resources for immigrant mothers, especially those of refugee status was also identified by health professionals (Hufton & Raven, 2016).

## 4 DISCUSSION

This mixed‐methods systematic review presents a synthesis of the evidence on the factors influencing breastfeeding practices among mothers of African origin who have migrated to a HIC. Breastfeeding initiation was high among the African mothers in this review but reduced considerably within the first few weeks. Data available on mode of feeding adopted and the duration varied widely across studies. However, it was clear that breastfeeding was fairly common among the mothers studied, but EBF for up to 6 months, according to the WHO recommendation (WHO, 2016), was very rarely practiced. African immigrant mothers, however, were more likely to breastfeed when compared with native mothers of the host country. Prolonged breastfeeding for up to 1 year was equally practiced by many of the African mothers but could range from as little as 3 months to 3 years. The evidence also showed that African mothers had strong beliefs and attitudes towards breastfeeding, which influenced their breastfeeding practices and decisions in both positive and negative ways. In particular, African mothers were more likely to supplement breastfeeding with formula milk after migration for various reasons including reduced familial support after childbirth, the different culture and lifestyle experienced in the new environment, increased financial freedom, influence of family and friends, absence of adequate information, and their cultural perceptions of the health of an infant. Health professionals agreed with the mothers that their cultural beliefs around breastfeeding, friends and family opinions, and language barriers were factors influencing breastfeeding practices.

Findings from this review showed that African immigrant mothers consider supplementing early with formula milk as an adoption of the Western culture. However, it is uncertain that EBF is truly a common practice in their home countries owing to their beliefs about giving water within the first week and discarding colostrum. A meta‐analysis of 29 countries in Sub‐Saharan Africa revealed that both EBF and predominant breastfeeding were practiced by less than half of all mothers (Issaka, Agho, & Renzaho, 2017). Similarly, a study conducted in three African countries showed that EBF rates are low as a result of cultural practices such as discarding colostrum and pre‐lacteal feeding (Engebretsen et al., 2014). Although EBF appears not to be widely practiced in African countries, formula feeding was not considered a common practice (Issaka et al., 2017). Breastfeeding in public was considered a factor influencing breastfeeding practices in HICs, and the mothers expressed the embarrassment and stigma associated with breastfeeding in public. However, some mothers found strategies to continue breastfeeding despite the inconvenience, whereas others resorted to formula feeding.

A review of the literature on the attitudes towards breastfeeding showed that some countries in Northern Europe and North Africa are beginning to accept public breastfeeding, whereas contrary to popular beliefs, some countries in Africa and Asia are discouraging of the practice (Komodiki, Kontogeorgou, Papastavrou, Volaki, & Genitsaridi, 2014). Similarly, a lack of adequate support in HICs influenced the breastfeeding practices of the mothers in this review and resulted in a change of roles for both fathers and mothers. The fathers got more involved in household responsibilities, whereas mothers had to work to support the family income. Friends and family were also usually too busy to offer much support, and when the mothers received the support they required, it became challenging to navigate information from varying sources such as health professionals and family members. Infants' grandmothers and other older mothers within the family were the most influential in the decisions around breastfeeding, similar to findings from studies of other immigrant populations (Lindsay, Le, & Greaney, 2017), and the mothers did not feel confident enough to go against their suggestions.

The health professionals' findings were in agreement with what the mothers reported, and they expressed that they faced challenges working with African mothers due to their cultural beliefs and the influence of their family members whose advice and opinion were more valued. This is contrary to the findings from a review of the belief, attitudes, and practices of Chinese immigrant mothers in developed countries (Lindsay et al., 2017) where the opinions of health professionals was found to be highly valued by the mothers. Language barrier was equally a major challenge highlighted by the health professionals, and the use of interpreters was sometimes perceived as inappropriate due to gender concerns.

### 4.1 Quality of the evidence

The majority of the included studies had fair (*n* = 19) or poor (*n* = 3) ratings compared with 12 good‐rated studies, suggesting that the findings should be interpreted with caution. Among fairly rated studies, most (*n* = 12) were rated so due to missing information on sampling strategy suggesting that there may be existing bias in the populations within individual studies and certain groups of individuals may have been over‐ or under‐represented. Additionally, not adjusting for confounding variables and an absence of description of the analysis process was common among fair‐rated studies (*n* = 8), and their results should be interpreted cautiously. Three (Kolanen et al., 2016; Textor et al., 2013; Tyler et al., 2014) of the included studies were rated as poor, and although they have been included in the synthesis, there was evidence from other better rated studies to support their findings. Despite the variability of data reported across studies and the poor rating of some studies, it was still possible to make some inferences from the data presented.

### 4.2 Strengths and limitations

The methodological strengths of this review include the robustness of the searches that involved searching 12 databases, reference and citation searches of all included studies, and percentage double screening, data extraction, and quality assessment. Adopting an integrated mixed‐methods approach meant that all elements of the data presented in individual studies were presented in the analysis and adopting elements of framework synthesis helped to capture existing evidence while identifying the gaps in the evidence. All studies meeting the inclusion criteria were included irrespective of the year or publication or study design (qualitative or quantitative), with the majority of quantitative studies being cohorts, strengthening the available evidence for the review.

However, limiting included studies to only studies published in the English language or with existing translations implies that some relevant information may have been missed from the review. Additionally, it was not possible to contact all the authors where required either because the authors did not respond to several attempts at making contact or author contact information could not be retrieved. One potentially relevant quantitative study (Merten, Wyss, & Ackermann‐Liebrich, 2007) was not included in the review because the data presented in the study was unclear, and the authors could not be reached. The heterogeneity of data reporting and outcomes assessed in individual quantitative studies made it impossible to pool the results into a meta‐analysis. The unclear description of the immigration status of the population of study in many of the individual studies (e.g. African‐American without indication of immigration history) meant that potentially relevant studies may have been excluded, and some studies which included African populations did not present a separate analysis on Africans. Furthermore, all Africans (including refugees and asylum seekers) have been reviewed as one population in this review, whereas there may be differences between sub‐Saharan Africa and North African populations and between voluntary migrants and migrants with refugee or asylum‐seekers status.

## 5 CONCLUSION

African mothers who have migrated to HICs are faced with challenges to their breastfeeding practices and experiences. Although these mothers appear keen to exclusively breastfeed, they are unable to, and additional support and information may be required to increase breastfeeding rates among African mothers living in HICs. An improved understanding of how migration influences breastfeeding, and how health professionals can better support African mothers who have migrated to HICs, may improve breastfeeding success. However, the contradictions in the beliefs and attitudes among the mothers show that there is no singular belief system among all African mothers and the approach to supporting mothers needs to be individualised. Future research should aim to explore the evidence gaps identified in this review such as studying a more varied population of African mothers including those with refugee and non‐refugee status.

## CONFLICTS OF INTEREST

The authors declare that they have no conflicts of interest.

## CONTRIBUTIONS

Conceptualisation: AO, NE, and JR; Data curation: AO, NE, and JR; Formal analysis: AO; Investigation: AO, NE, and JR; Screening and data collection: AO, NE, LN, WA, and JR; Project administration: AO, NE, and JR, Supervision: NE and JR; Validation: AO, NE, LN, WA, and JR; Visualisation: AO; Writing original draft: AO; Reviewing and editing drafts: AO, NE, LN, WA, and JR.
